# Categorized and individualized behavioral phenotyping approaches for control and triple-hit schizophrenia-like model rats in acute and chronic reward-based systems: A pilot study

**DOI:** 10.1371/journal.pone.0328460

**Published:** 2025-08-07

**Authors:** Zsofia Hernadi, Laszlo Kormoczi, Leatitia G. Adlan, Gabriella Kekesi, Alexandra Büki, Peter Liszli, Laszlo G. Nyúl, Gábor Braunitzer, Gyongyi Horvath

**Affiliations:** 1 Department of Physiology, Albert Szent-Györgyi Medical School, University of Szeged, Szeged, Hungary; 2 Department of Image Processing and Computer Graphics, Faculty of Science and Informatics, University of Szeged, Szeged, Hungary; 3 Sztárai Institute, University of Tokaj, Sárospatak, Hungary; Northwestern University Feinberg School of Medicine, UNITED STATES OF AMERICA

## Abstract

A wide variety of acute test procedures are used for the behavioral characterization of schizophrenia rodent models, but prolonged observation in home-cage systems remains underexplored. This study aimed to profile the behavior of control (Wistar) and triple-hit schizophrenia-like (Wisket) model rats under two reward-based test conditions: the acute Ambitus test, a simple reward-collection task, and the delay-discounting paradigm in the HomeManner system, designed for extended observation periods. Wisket rats exhibited significant behavioral impairments compared to Wistar rats in the Ambitus test. However, in the HomeManner system, no significant group differences were observed at the cohort level due to heterogeneous individual behavioral patterns. Specifically, only half of the rats explored both food-providing trays, while others focused on one or ignored both. Subgroup analysis, based on tray activity patterns, revealed that Wisket rats displayed impaired cognitive processes and greater intolerance to delayed food rewards. Furthermore, personalized analysis indicated lower behavioral variability within the Wisket group compared to controls. The absence of a strong correlation between performance in the Ambitus and HomeManner tests suggests that these assessments capture distinct behavioral characteristics. This pilot study provides an extended behavioral phenotyping of rat substrains under both acute and chronic conditions. The automated HomeManner system demonstrates potential as a valuable tool for prolonged behavioral assessments. Importantly, this study underscores the value of categorized and personalized analyses in revealing behavioral differences that may not be apparent in group-level comparisons, thereby enhancing the translational relevance of preclinical research.

## Introduction

A wide variety of acute test procedures are employed to analyze different behavioral aspects in healthy animals and disease models, including schizophrenia. However, these assays have notable limitations, such as the acute stress induced by test-related procedures and their unsuitability for prolonged observations [1,2]. Automated home-cage systems with environmental enrichment address these issues by minimizing human interaction, potentially improving test reproducibility, and providing animals with more naturalistic conditions [3]. These systems allow the detection of spontaneous behaviors in an undisturbed setting, enabling more comprehensive behavioral assessments compared to traditional methods. Recently, several home-cage activity systems have been developed to facilitate continuous, long-term monitoring of locomotion, feeding behavior, cognitive function, and drug-induced behavioral changes. Some systems rely on video monitoring (e.g., PhenoTyper), while others use infrared sensors to track individual animals (e.g., PhenoMaster). PhenoTyper is commonly utilized for studying locomotor activity in individually housed rodents [4,5], whereas IntelliCage and PhenoWorld can monitor the behavior of microchipped animals in group-housed settings [6–8]. To date, these complex behavioral systems have primarily been applied to healthy animals, with only one study investigating behavioral alterations in a schizophrenia mouse model under standard (small) home-cage conditions [9].

Schizophrenia is a mental disorder characterized by positive and negative symptoms, as well as cognitive impairments. Animal modeling in neuroscience is particularly challenging due to the significantly greater complexity of the human brain compared to animal brains. Additionally, many preclinical models fail to account for the chronic nature of neuropsychiatric disorders or the critical role of gene-environment interactions in their development and symptomatology. To address these limitations and create a model with high construct validity for schizophrenia, our laboratory developed the Wisket rat substrain from the Wistar strain. This “multiple hit” model combines environmental (post-weaning social isolation), pharmacological (treatment with the N-methyl-d-aspartate [NMDA] receptor antagonist ketamine), and genetic (selective breeding based on behavioral phenotype for more than 40 generations) manipulations [10–12]. Acute behavioral tests have demonstrated that Wisket animals display a range of disturbances associated with schizophrenia, including impairments in sensory gating, social behavior, pain sensitivity, locomotor and exploratory activities, as well as cognitive functions such as learning and memory [10,12,13]. Supporting the model’s predictive validity, previous studies have shown that cognitive training improves these deficits, and the antidiabetic drug metformin mitigates the behavioral side effects of the antipsychotic clozapine [11,14]. While no single symptom observed in animal studies is specific to schizophrenia, the combination of complex behavioral patterns, along with the construct and predictive validity of the Wisket model, supports its suitability for preclinical research into this disorder.

In addition to these symptoms, impaired self-control or impulsiveness during decision-making is a common, though nonspecific, feature of schizophrenia. In delay discounting tasks, where subjects choose between a large delayed (LD) reward and a small immediate (SD) reward, both schizophrenia patients and animal models frequently exhibit similar impairments [15–17]. Koot et al. studied impulsivity in control rats housed in standard cages using the delay discounting test [18]; however, no studies have yet explored this paradigm in larger cages with environmental enrichment and minimal human interaction.

Subject heterogeneity is a well-documented phenomenon, particularly in schizophrenia, and it often leads to conflicting results in group-level analyses, making it difficult to identify reliable markers for the disease [19]. This heterogeneity arises from complex gene-environment interactions, variability in symptom presentation, and differences in treatment responses. Personalized or precision medicine is an approach that addresses these challenges by tailoring therapies to individual patients, aiming for greater efficacy. Similarly, in preclinical research, some studies have begun to emphasize the importance of individual differences in assessing disease-related functional alterations [1,20,21].

The aim of this pilot study was to evaluate the utility of our newly developed system, HomeManner—a large, environmentally enriched cage designed to reduce human interaction and support the delay discounting paradigm—by applying comprehensive categorized and personalized behavioral phenotyping to control and triple-hit Wisket model rats. We hypothesized that data collected under these relatively stress-free conditions over an extended period, even with a small sample size, could offer a reliable translational approach for advancing personalized medicine.

## Materials and methods

### Animals

All experiments were approved by the Hungarian Ethical Committee for Animal Research (RN: XIV/1248/2018) and conducted in compliance with the EU Directive 2010/63/EU and the Animal Research: Reporting of In Vivo Experiments (ARRIVE) guidelines 2.0.

Male control and Wisket rats (N = 9 per group) were used in the study. The animals were maintained under a 12-hour light/dark cycle at a controlled temperature of 22 ± 1°C and humidity of 55 ± 10%. Based on previous studies, Wisket rats were housed individually starting after weaning at 3 weeks of age for 28 days to produce social isolation as an environmental hit. During social isolation, the individuals were placed in separate boxes, but within visual, auditory and olfactory distance according to the regulations to alleviate suffering. Then, they received intraperitoneal ketamine treatment (30 mg/kg/day for 5 days, Calypsol, Gedeon Richter Plc., Hungary) during the second week of isolation, as a pharmacological hit [14,22]. To alleviate animals’ suffering during repeated intraperitoneal injections of ketamine, the minimally necessary restraint was applied. Following this period, they were re-housed in groups (2–3 per cage) and allowed a one-week recovery period without further interventions. Resocialization did not lead to aggressiveness or injury in these animals. Control animals were socially housed throughout and did not receive ketamine or vehicle injections to prevent them from any stress. At the end of the experimental protocol, the animals were humanely sacrificed by decapitation using a rat guillotine 24 hours after the final injection.

At 10 weeks of age, all animals participated in an acute behavioral test (Ambitus), as described in previous studies [10,11]. Briefly, food restriction was applied 48 hours prior to the test, with water available ad libitum, to ensure adequate motivation for task performance. Two types of tasks were conducted: Task 1 (Trials 1–2, all boxes baited with rewards) in the morning, followed three hours later by Task 2 (Trials 3–4, only the inner boxes baited). Each rat completed two sessions (two trials per session, separated by one minute) three hours apart.

At least one week after the Ambitus test, the animals were moved to the testing room for two days under food restriction to enhance their motivation to consume the rewards in the HomeManner system. The animals were then assigned to one of six cages in a pseudo-randomized manner and housed individually without visual contact for 13 days. Both groups were evenly distributed across three experimental rounds to ensure nine animals per group.

On Day 1 of the experiment, the animals were placed in the large cages between 9:00 and 10:00 AM without standard food, but with both pellet dispensers filled (12 g + 4 g = 16 g). For the following days, 10 g of standard food was provided daily on the first floor near the pellet-providing trays. This setup maintained a moderate food restriction throughout the HomeManner experiment; however, the animals could meet their nutritional requirements by consuming pellets from the dispensers.

At the start of the experiment, no delay was applied to the LD side until the animals demonstrated a preference for the LD reward. Once a preference was established (defined as at least 10% above random preference), a 10-second delay was introduced on the LD side. This delay was gradually increased by 10 seconds each day, depending on the animals’ preference, following a protocol from a previous study [18]. Apart from the introduction of delay, no other parameters were modified throughout the experiment.

Body weight was measured at the beginning and end of the HomeManner experiment. Fluid consumption was monitored twice a week, replaced with fresh water, and adjusted relative to body weight (relative fluid consumption). Pellet consumption was recorded daily, and the dispensers were refilled to maintain 12 g for the LD side and 4 g for the SD side. Standard food consumption was measured at the conclusion of the experiment. Disturbance to the animals during the testing period was minimized; the experimenter typically entered the room once daily (except in cases of equipment malfunction) to verify the functionality of the apparatus, check the animals’ health, refill dispensers, provide standard food, and replace drinking water.

#### Ambitus system.

**Fig 1:**
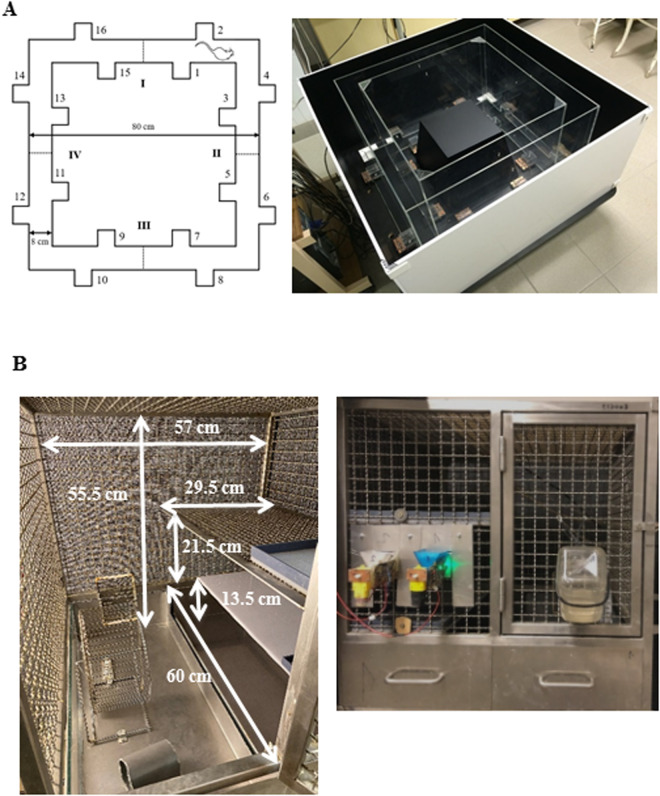
The structure and view of the Ambitus apparatus (A) and the HomeManner system (B). The image of the Ambitus apparatus is sourced from the manufacturer’s website: http://www.deakdelta.hu/ambitus.html.

The Ambitus apparatus (Deák Delta Ltd., Hungary) is a rectangular corridor made of clear Plexiglas with a black floor (Fig 1A) [20]. The corridor contains four side boxes on each wall (16 boxes in total) designed to hold food rewards (puffed rice, 20 mg). Infrared beams are used to detect exploratory activity at each side box and locomotor activity in the midpoint of the corridor, with a time resolution of 1 ms. The analyzed parameters are listed in Table 1 (under Outcomes and Statistical analysis). At the start of each trial, food rewards were placed in the boxes, and the rats were positioned at the starting point (Fig 1A); the experimenter then left the room immediately. The animals were given 300 seconds to explore the corridor and collect the food rewards. The apparatus was cleaned with 70% alcohol between trials to maintain hygiene.

**Table 1:** Summary of measured and derived behavioral parameters with units and definitions.

Category	Parameter	Unit	Definition/Calculation
General	Water intake	mL/kg/day	Amount of water consumed per body weight.
	Food intake	g/kg/day	Amount of food consumed per body weight.
	Body weight change	%	Percentage change in body weight.
Ambitus	Locomotor activity (LOCO/TOT)	number	Number of corridor entries within 5 minutes.
	Locomotor activity (LOCO/BEF)	number	(Number of entries × 300)/ Eating time (s).
	Exploratory behavior (EXPL/TOT)	number	Total box visits within 5 minutes.
	Exploratory behavior (EXPL/BEF)	number	Number of box visits before reward collection.
	Internal visits (EXPL/BEF_I)	number	Subset of EXPL/BEF categorized as internal box visits.
	External visits (EXPL/BEF_E)	number	Subset of EXPL/BEF categorized as external box visits.
	Locomotion-independent exploration (Indep_EXPL/BEF_E)	number	The ratio of external visits to locomotor activity until all rewards were collected.
	Locomotion-independent exploration (Indep_EXPL/BEF_I)	number	The ratio of internal visits to locomotor activity until all rewards were collected.
	Learning capacity (L_C)	%	(Eating count × 300 × 100)/ (Number of rewards × Eating time (s)).
HomeManner	Impulsivity based on premature exploration	%	(E2 × 100)/ Total exploratory events.
	Impulsivity based on incorrect exploration	%	(E3 × 100)/ Total exploratory events.
	Delay time	seconds (s)	Interval between stimulus trigger and food delivery at LD side.
	Eating time	seconds (s)	Time taken to consume all pellets, capped at 24 hours (86,400 s).
	Hourly exploratory activity	number/hour	(Total exploration events × 3600)/ Eating time (s).
	Accuracy	%	(E1 × 100)/ (E1 + E2 + E3).
	Prematurity	%	(E2 × 100)/ (E1 + E2).
	Incorrect response ratio	%	(E3 × 100)/ (E1 + E3).
	Learning capacity (LC)	%	(Eating count × 86,400 × 100)/ (Number of rewards × Eating time (s)).

E1: adequate exploration; E2: premature exploration; E3: incorrect exploration

#### HomeManner system.

The HomeManner system consists of six operant cages located in a separate room with house lighting outside the cages, maintained under standard environmental conditions (Fig 1B). Each cage measures 57 × 60 × 55.5 cm and features three levels. The sidewalls and top of the cages are constructed from steel wire grid (Fig 1B). Since a structured cage environment is considered more beneficial for rodents than a large, open floor area [23], each cage is divided into two sections: one with a single floor and the other with three levels. The animals can access all levels by climbing the grid walls.

In the single-floor section, a play area is created to promote the animals’ well-being and provide sensory and motor stimulation. This area is equipped with a running wheel (for voluntary exercise), an abacus, and a plastic tube for environmental enrichment. At the front of the cage, a bottle provides free access to drinking water, accessible from the play area (Fig 1B).

The first level of the three-floor side serves as a shelter (28.5 × 60 × 11 cm) with bedding material (sawdust, 3–4 cm thick) where the animals can also hide marbles. Above the shelter, the second and third levels are constructed from opaque plastic and steel grid, respectively. On the second floor, 20 cm from the drinking bottle, two food dispensers are attached. These dispensers consist of external food reward containers equipped with small electric motors that deliver 45 mg food pellets (F0021, BioServ, Frenchtown, NJ, US) into two trays (12 × 20 mm) spaced 7 cm apart, which are accessible inside the cage. LED lights are attached near the containers to signal reward availability. The trays are equipped with sensors (infrared light beams) to detect reward delivery, eating behavior, and the activity of the animals at the trays. Interactions with the trays are termed exploration. The panel is connected via an interface to a PC in a separate room, where specific software (developed by P.L.) controls food delivery and records all events at both trays (for the parameters, see Table 3 in the Results section). Data are subsequently extracted and analyzed using another software program created by L.K.

**Table 3:** Statistical summary of the parameters analyzed in the Ambitus system. The numbering corresponds to the numbering of Fig 6.

Parameters: Definition/Calculation	Results with all trials			Results with all subgroups		
	Group	Trial	Group/ Trial	Group	SG	Group/SG
1. LOCO/TOT: Locomotor activity (n): number of entries into corridors up to 5 min	12.16;(1,64) <0.001	20.25;(3,64) <0.001	4.77;(3,64) <0.005	9.24;(1,28) <0.01		
2. LOCO/BEF: Locomotor activity before collecting the rewards: (number of entries into corridors)x300/ET)	11.98;(1,64) <0.001	17.94;(3,64) <0.001	4.03;(3,64) <0.05	8.31;(1,28) <0.01		
3. EXPL/TOT: Overall number of box visits up to 5 min	27.64;(1,64) <0.001	3.61;(3,64) <0.05	7.62;(3,64) <0.001	73.26;(1,28) <0.001		
4. EXPL/BEF: (Overall number of box visits until all reward collection)x300/(ET)	19.29;(1,64) <0.001	4.04;(3,64) <0.05	5.37;(3,64) <0.001	30.32;(1,28) <0.001		
5. EXPL/BEF_E: (number of external box visits until all reward collection)x300/(ET)	11.35;(1,64) <0.005			38.51;(1,28) <0.001		5.19;(3,28) <0.001
6. EXPL/BEF_I: (number of internal box visits until all reward collection)x300/(ET)	19.22;(1,64) <0.001	7.82;(3,64) <0.001	6.19;(3,64) <0.001	21.80;(1,28) <0.001		
7. Indep_EXPL/BEF_E: locomotion independent exploration (number of external box visits until all reward collection)/(number of locomotor activity until all reward collection)		8.26;(3,64) <0.001		16.09;(1,28) <0.001	3.11;(3,28) <0.05	6.45;(3,28) <0.005
8. Indep_EXPL/BEF/I: locomotion independent exploration (number of internal box visits until all reward collection)/(number of locomotor activity until all reward collection)	4.97;(1,64) <0.05	5.36;(3,64) <0.005		18.22;(1,28) <0.001		5.55;(3,28) <0.005
9. Learning capacity (L_C; %): [(Eating count)x(300)x100]/ [number of rewards)x(ET)]	19.10;(1,64) <0.001	9.53;(3,64) <0.001	6.69;(3,64) <0.001	19.84;(1,28) <0.001		

ET: eating time; Numbers within the cells mean: F value; (Degree of Freedom); P value

At the beginning of the experiment, the pellet dispensers were filled with 12 g or 4 g of pellets at the trays designated as the large-dose (LD) and small-dose (SD) sides, respectively, and these assignments remained fixed throughout the study. When the animals were placed in their cages, the LED lights were switched on, and the dispensers delivered 3 or 1 pellets (on the LD and SD sides, respectively) to the trays, following a paradigm based on a previous study [24]. As the animals consumed pellets from any tray, the LED light on that side was turned off, and no reward was available for 20 seconds (inter-trial interval). After the inter-trial interval, the LED light turned back on, and when exploration was registered (trigger stimulus), food pellet(s) were dispensed into the trays. This setup ensured that the animals’ activity at the trays initiated a trial.

Rats were not restricted by time to initiate trials, allowing their activity at the trays to be entirely voluntary throughout the investigation period. Although the trays were accessible 24 hours a day, once the animals consumed all the food allocated for the day (12 g + 4 g by dispensers), the LED lights were turned off until the next day. Between 9 and 10 AM daily, the dispensers were refilled, trays were rebaited with pellets (3 or 1), and the LED lights were switched back on. In cases of hardware malfunctions, the LED lights automatically switched off.

### Outcomes and statistical analysis

The Ambitus and HomeManner systems were utilized to assess exploratory and eating behaviors in animals, facilitating the calculation of multiple behavioral parameters. The study examined distinct types of exploration and eating activities, categorized animals into subgroups, and evaluated behavioral metrics.

In the HomeManner system, three types of exploration were identified. Adequate exploration, referred to as E1, involved activity triggered by the stimulus and occurring between reward delivery and consumption. Premature exploration, identified as E2, occurred during the inter-trial interval and was indicative of impulsivity. Incorrect exploration, denoted as E3, included activity between the stimulus and reward delivery, which could occur with “no” delay, typically involving 1–2 seconds for reward delivery. Ratios of E2 and E3 to the total exploratory events were used as measures of impulsivity [25].

Animals in the HomeManner system were categorized into four subgroups based on exploratory activity patterns. Subgroup 1 (SG1) consisted of animals (N_control_ = 5; N_Wisket_ = 4) exhibiting activity at both reward-providing trays. Subgroup 2 (SG2) included animals (N_control_ = 1; N_Wisket_ = 2) active only at the larger dose (LD) tray. Subgroup 3 (SG3) comprised animals (N_control_ = 2; N_Wisket_ = 1) active only at the smaller dose (SD) tray. Subgroup 4 (SG4) represented animals with no activity at either tray (N_control_ = 1; N_Wisket_ = 2). Data from SG4 were excluded from detailed analyses due to insufficient activity. For further analyses, data were normalized to relative numbers of exploration events and limited to days when technical errors were absent.

General observations included water intake relative to body weight (mL/kg/day), food intake relative to body weight (g/kg/day), and changes in body weight (%). Locomotor and exploratory activities were extensively assessed in the Ambitus test. Locomotor activity (LOCO/TOT) was quantified as the number of entries into corridors within five minutes. To evaluate behavior before reward collection, locomotor activity (LOCO/BEF) was calculated as the number of entries multiplied by 300 and divided by the eating time (s). Exploratory behavior included the total number of box visits within five minutes (EXPL/TOT) and the number of box visits before reward collection (EXPL/BEF), normalized using a similar calculation. Exploratory visits were further categorized into internal (EXPL/BEF_I) and external (EXPL/BEF_E) visits, with additional calculations applied to assess locomotion-independent exploration (Indep_EXPL/BEF_E and Indep_EXPL/BEF_I), such as dividing the number of external or internal visits by the locomotor activity until all rewards were collected. Learning capacity (L_C), expressed as a percentage (%), was calculated by multiplying the eating count by 300 and 100, and dividing the result by the product of the number of rewards and eating time (s).

In the HomeManner system, delay time (s) was recorded as the interval between the stimulus trigger and food delivery at the LD side. Eating time, defined as the time required to consume all pellets, was capped at 24 hours (86,400 s). Exploratory activity was expressed as the hourly number of explorations (Hourly Exploratory Activity) and calculated by multiplying the total exploration events by 3600 and dividing by the eating time (s). Accuracy (%), defined as the percentage of E1 events out of the total exploratory events, was calculated as the product of E1 and 100 divided by the sum of E1, E2, and E3. Prematurity (%), indicating the proportion of E2 events relative to E1 and E2 combined, was calculated similarly by multiplying E2 by 100 and dividing by the sum of E1 and E2. Incorrect exploration ratios (Incorrect Response Ratio, %) were calculated by dividing E3 by the sum of E1 and E3 and multiplying by 100. Learning capacity (LC, %) in this system was calculated by multiplying the eating count by 86,400 and 100, then dividing by the product of the number of rewards and eating time (s).

Inter-individual variability across behavioral parameters was assessed using z-scores and heatmaps. The z-scores were calculated by subtracting the population mean from each individual value and dividing by the standard deviation of the population [26]. Heatmaps were utilized to visualize the variability and subgroup patterns observed in the Ambitus and HomeManner tests. Table 1 summarizes all measured and derived parameters.

**Fig 2:**
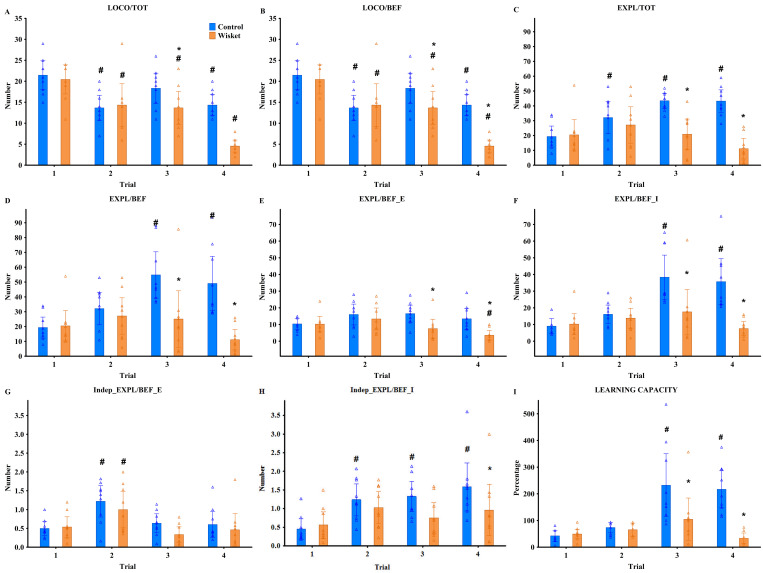
Behavioral characterization of animals in the Ambitus test across four trials at the group level, with triangles representing individual values. Definitions, abbreviations, and parameter calculations are provided in Table 3. Symbols indicate significant differences (p < 0.05): (*) vs. control, (#) vs. trial 1.

**Fig 3:**
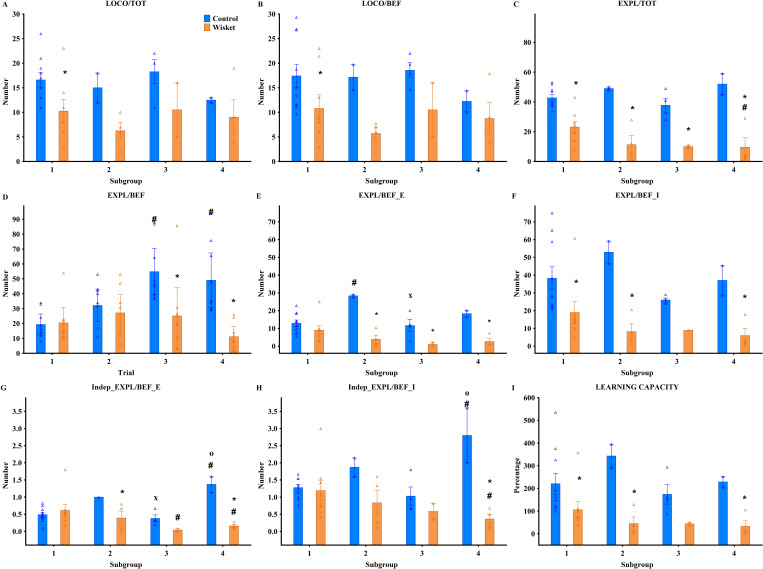
Behavioral characterization of animals in the Ambitus test at the subgroup level, including pooled data from Trials 3 and 4, with triangles representing individual values. Definitions, abbreviations, and parameter calculations are provided in Table 3. Symbols indicate significant differences (p < 0.05): (*) vs. control, (#) vs. SG1, (x) vs. SG2, (o) vs. SG3.

**Fig 4:**
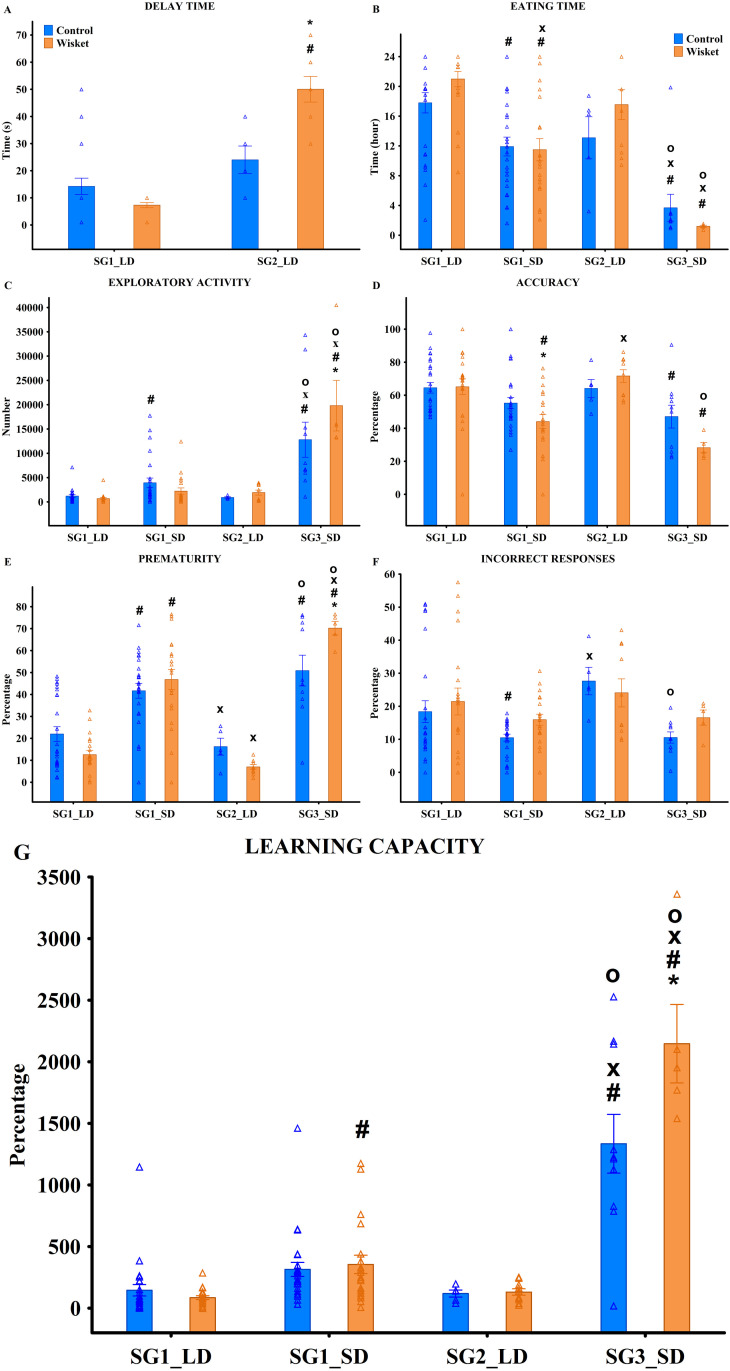
Behavioral characterization of animals in the HomeManner system across different subsets, with triangles representing individual values. Definitions and parameter calculations are provided in Table 4. Symbols indicate significant differences (p < 0.05): (*) vs. control, (#) vs. SG1_LD, (x) vs. SG1_SD, (o) vs. SG2_LD.

**Fig 5:**
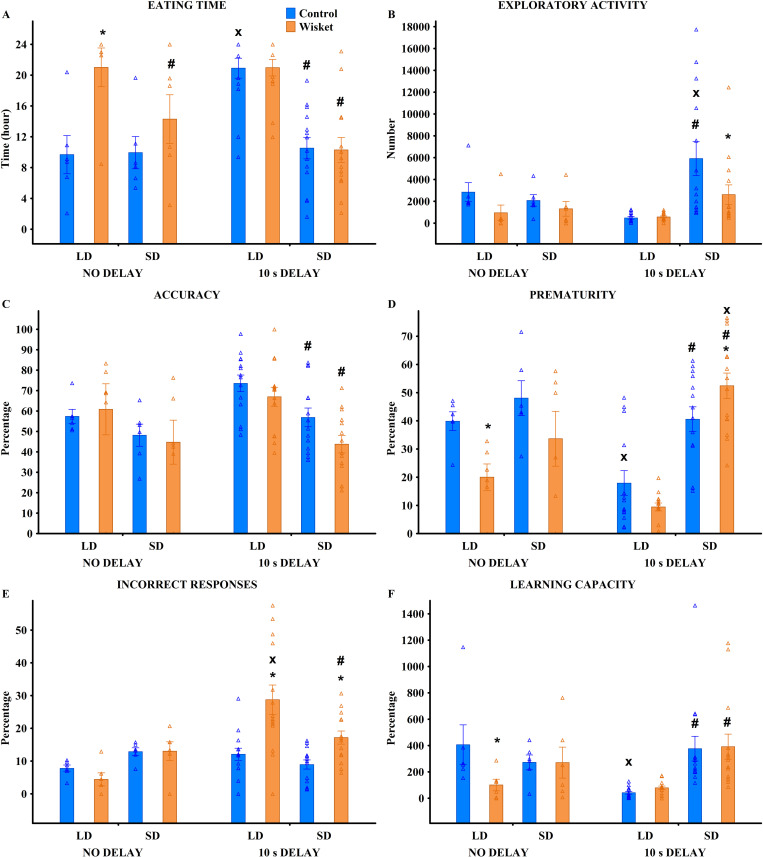
Behavioral characterization of animals in the HomeManner system within SG1 under “no” or 10-second delay conditions, with triangles representing individual values. Definitions and parameter calculations are provided in Table 3. Symbols indicate significant differences (p < 0.05): (*) vs. control, (#) by side, and (x) by delay.

Statistical analyses were conducted to evaluate group-level and subgroup-level differences. General observations and covariance data were analyzed using one-way ANOVA, while factorial ANOVA was applied to behavioral parameters across subgroups and trials. In the Ambitus test, the factors were group (control vs. Wisket) and either trial (Trial 1–4; Fig 2) or subgroup (SG1–SG4; Fig 3). For the HomeManner system, the factors included group (control vs. Wisket) and subsets (SG1_LD, SG1_SD, SG2_LD, SG3_SD; Fig 4). In cases where SG1 subgroups with “no” or 10-second delay were analyzed separately, the factors were group, side (LD vs. SD), and delay (Fig 5). Post hoc analyses using Fisher’s LSD test were performed to evaluate the significance of various factors. Spearman correlations were used to explore relationships between delay times and exploratory parameters within specific subgroups, as well as to identify associations between metrics across the Ambitus and HomeManner systems. Data are reported as means ± S.E.M., with statistical significance set at p < 0.05. Statistical analyses were performed using STATISTICA 13.5.0.14 (TIBCO Software Inc., USA). This integrated approach ensured a comprehensive and precise evaluation of the behavioral activities across systems.

## Results

### General observations

Table 2 summarizes the results of the general observations. Relative fluid intake did not differ significantly between the two groups, as determined by one-way ANOVA. All animals consumed the available standard food (10 g/day, approximately 60% of the daily food requirement) [27], and no significant differences in total food consumption were observed between the control and Wisket rats. However, significant differences were found between the subgroups. Specifically, animals that ate only from the SD trays (SG3) or did not eat from the trays at all (SG4) consumed significantly less food compared to the other subgroups.

**Table 2:** Statistical results of general observations analyzed using one-way ANOVA.

Parameters	Mean±SEM		Effect	Mean±SEM				Effect: F;(df);P
	Control	Wisket		SG1	SG2	SG3	SG4	
Water intake relative to body weight (mL/kg/day)	72 ± 9.2	72 ± 3.8	NS	66 ± 3.1	78 ± 8.4	64 ± 4.5	92 ± 26.1	NS
Food intake relative to body weight (g/kg/day)	44 ± 3.3	44 ± 3.5	NS	49 ± 2.3	51 ± 3.6	33 ± 1.2	32 ± 1.2	11.78;(3,14); < 0.001
Body weight change (%)	−3.6 ± 2.77	−0.7 ± 3.19	NS	2.9 ± 2.05	2.1 ± 3.21	−8.8 ± 0.40	−15.0 ± 2.96	10.15;(3,14); < 0.001

NS: non-significant; SEM: standard error of mean; SG: subgroup

Changes in body weight were correlated with food consumption. Both groups experienced slight weight loss by the end of the HomeManner test, with significant differences observed between the subgroups.

### Ambitus test

For most behavioral parameters measured in the Ambitus test (Fig 1A), factorial ANOVA revealed significant effects of group, trial, and their interaction (Table 3; Fig 2A–I). Compared to their parent strain, Wisket rats exhibited reduced locomotor, exploratory, and eating activities, as well as impaired learning abilities, particularly during Trials 3 and 4. Consequently, pooled data from these trials were analyzed at the subgroup level (Fig 3A–I). ANOVA identified significant subgroup effects only for locomotion-independent exploration of the external boxes (Table 3; Fig 3G), driven by the high values observed in SG2 animals in both groups.

### HomeManner system

Regarding the overall daily activity of control and Wisket animals (N = 8 and N = 7, respectively) at their active side, the animals collected an average of 128 ± 7.6 pellets daily from the reward dispensers and exhibited 944 ± 145.7 daily exploration events.

Heterogeneous behavioral patterns were observed at the trays, limiting the introduction of delay to the SG1 and SG2 subgroups. In these subgroups, significant effects of group, subgroup, and their interaction were identified (Table 4; Fig 4A). Specifically, Wisket animals in SG1 required significantly shorter delays, while those in SG2 required significantly longer delays compared to control animals.

**Table 4:** Statistical summary of the parameters analyzed in the HomeManner system. The numbering corresponds to the numbering of Fig 6.

Parameters: Definition/Calculation	Results with all subsets at active sides			Results within SG1 with “no” or 10 s delays					R value
	Group	SS	Group/ SS	Group	Side	Delay	Group/Delay	Side/ Delay	
10. Delay time: Delay between trigger and food delivered at LD side	6.29; (1,56) <0.05	47.63; (1,56) <0.001	18.78; (1,56) <0.001						
11. Eating time (ET): Time up to all pellets were consumed (cut-off: 24 h = 86400 s)		29.99; (3,112) <0.001		8,43; (1,72) <0.005	26.54; (1,72) <0.001		8.81; (1,72) <0.005	7.46; (1,72) <0.01	C/LD:0.57
12. Exploratory activity: Hourly number of explorations up to the collection of rewards: =(E_Tot*3600)/ET		37.17; (3,112) <0.001	2.94; (3,112) <0.05	3.99; (1,72) <0.05	5.84; (1,72) <0.05			7.19; (1,72) <0.01	
13. Accuracy (%): =(E1*100)/ (E1 + E2 + E3)		12.29; (3,112) <0.001			13.73; (1,72) <0.001				
14. Prematurity (%): =(E2*100)/(E1 + E2)		43.66; (3,112) <0.001	3.73; (3,112) <0.05	4.46; (1,72) <0.05	35.83; (1,72) <0.001		6.65; (1,72) <0.05	9.01; (1,72) <0.005	C/LD: −0.44 W/LD:-0.50
15. Incorrect response ratio (%): =(E3*100)/(E1 + E3)		4.89; (3,112) <0.005		6.05; (1,72); <0.05		10.74; (1,72); <0.005	10.18; (1,72) <0.005	10.39; (1,72) <0.005	C/LD: 0.73 W/LD: 0.68
16. Learning capacity (%): [(Eating count)x(86400)x100]/ [number of rewards)x(ET)]	7.35; (1,112) <0.01	84.77; (3,112) <0.001	6.00; (3,112) <0.001		7.74; (1,72) <0.01			6.17; (1,72) <0.05	

EXPL: exploration; C: control; NR: number; W: Wisket; LD: Large dose side; E1: adequate exploration, (i.e., trigger stimulus and explorations between the reward supplied and eaten). E2: premature exploration during the inter-trial interval. E3: incorrect exploration (exploration between trigger stimulus and reward supplied; including delays, as well). ET: eating time

Numbers within the cells mean: F value; (Degree of Freedom); P value; SS: subset; R value: significant correlation between delay and all the other behavioral parameters in SG1.

Factorial analysis of eating time revealed significant effects of subsets (Fig 4B; Table 3). Animals in SG1 exhibited shorter eating times at the SD side compared to the LD side in both groups, consuming all rewards more quickly on the SD side. In contrast, animals in SG3 consumed all pellets within a few hours exclusively from the SD side.

A separate analysis of SG1 animals with “no” delay or a 10-second delay showed significant effects of group, side, and their interactions (Table 4; Fig 5A). Post hoc analysis indicated that Wisket animals had significantly longer eating times under the “no” delay condition at the LD side compared to controls. However, this group difference disappeared with the introduction of a 10-second delay, which resulted in shorter eating times at the SD side compared to the LD side in both groups.

In terms of exploratory activity, the highest number of explorations was recorded in the SG3_SD subset of Wisket animals (Table 4; Fig 4C). Within the SG1 subgroup under “no” or 10-second delay conditions, control animals with a 10-second delay exhibited the highest exploratory activity (Table 4; Fig 5B).

Exploration ratios, including accuracy, prematurity, and incorrect exploration ratios are indicators of cognitive impairments and impulsivity [28]. Analysis revealed significantly lower accuracy levels in Wisket animals at the SD side in SG1 compared to control animals. Furthermore, the SG3_SD subset displayed the lowest accuracy values among all subsets (Table 4; Fig 4D). In the SG1 subgroup under “no” or 10-second delay conditions, accuracy was lower at the SD side compared to the LD side in both control and Wisket animals when a 10-second delay was applied (Table 4; Fig 5C).

Regarding prematurity, it was significantly higher at the SD side compared to the LD side in both control and Wisket animals within SG1. Additionally, prematurity was significantly higher in Wisket animals compared to controls in the SG3_SD subset (Table 4; Fig 4E). Within the SG1 subgroup under “no” or 10-second delay conditions, Wisket animals with no delay exhibited lower prematurity levels than controls, particularly at the LD side. However, when a 10-second delay was introduced, prematurity decreased at the LD side but increased at the SD side, with a significantly greater increase in Wisket animals compared to controls (Table 4; Fig 5D).

For the incorrect response ratio, consistent with the potential delay at the LD side, this ratio was moderately higher at the LD side compared to the SD side (Table 4; Fig 4F). Within SG1 under “no” or 10-second delay conditions, a significantly higher incorrect response ratio was observed in Wisket animals at both sides under the 10-second delay compared to controls (Table 4; Fig 5E).

Animals in the SG3_SD subset exhibited significantly higher learning capacity (LC) compared to all other subsets, with Wisket animals showing significantly higher LC than controls within this subset (Table 4; Fig 4G). Additionally, in SG1, Wisket animals demonstrated significantly higher LC at the SD side compared to the LD side. Within the SG1 subgroup under “no” or 10-second delay conditions, LC was significantly lower in Wisket animals at the LD side compared to controls. However, with the introduction of a 10-second delay, LC became significantly higher at the SD side compared to the LD side in both groups (Table 4; Fig 5F).

Spearman correlation analysis within SG1 revealed significant correlations between delay time and eating time in the control group, as well as with prematurity and the incorrect response ratio in both groups at the LD side (Table 4, rightmost column). In terms of exploratory activity, no significant correlations were observed in the Ambitus and HomeManner tests.

### Analyses of inter-individual differences

**Fig 6:**
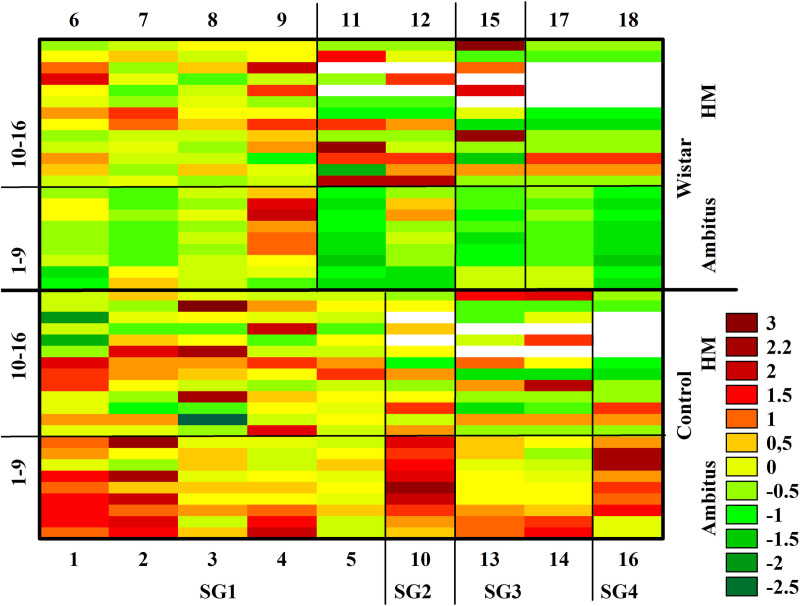
Complex heatmap of z-scores for the investigated parameters obtained in the Ambitus and HomeManner tests for both groups. The numbers of the animals are indicated along the lower horizontal axis for the control group and the upper horizontal axis for the Wisket group. Parameters: (Ambitus) 1. LOCO/TOT, 2. LOCO/BEF, 3. EXPL/TOT, 4. EXPL/BEF, 5. EXPL/BEF_E, 6. EXPL/BEF_I, 7. Indep_EXPL/BEF_E, 8. Indep_EXPL/BEF/I, 9. Learning capacity (L_C); (HomeManner) 10. Delay time, 11. Eating time (ET), 12. Exploratory activity, 13. Accuracy, 14. Prematurity, 15. Incorrect response ratio, 16. Learning capacity. See also Tables 3 and 4.

A personalized analysis of z-scores obtained from the Ambitus test, visualized using a heatmap, revealed high inter-individual differences among control animals but not in the Wisket group. These differences were most prominent in parameters measured up to the collection of rewards (1st–9th rows in Fig 6). This finding was supported by covariance values (Controls: 2.0 ± 0.26 vs. Wisket: 1.3 ± 0.13; P < 0.05).

The personalized analysis of HomeManner data showed slightly lower inter-individual differences in SG1 Wisket rats compared to controls regarding delay time (Fig 6). For example, a 50-second delay could be applied to one control animal (N4: 4th bar in the control group; Fig 6), while another control animal (N3: 3rd bar in the control group; Fig 6) did not learn to prefer the LD side, making delay introduction impossible for that animal. Additionally, the N1 control animal in SG1 demonstrated extremely high activity at the SD side, whereas all animals in SG3 displayed elevated levels of exploratory activity and learning capacity (LC) values.

## Discussion

This study analyzed control (Wistar) and triple-hit schizophrenia-like (Wisket) rats at the group, subgroup/subset, and individual levels under two conditions: behavioral assessment in the acute Ambitus test and prolonged automated behavioral testing using the HomeManner system. Consistent with previous findings, Wisket rats exhibited significant behavioral impairments in the Ambitus test [11,22], with low inter-individual variability. In contrast, data from the HomeManner system showed moderate group-level differences. However, analysis at the subgroup/subset level revealed lower learning abilities, greater impulsivity, and reduced inter-individual variability (as indicated by covariance values) in Wisket rats compared to controls. Moreover, no significant relationship was found between results from the Ambitus and HomeManner tests, suggesting that these methods assess distinct aspects of behavioral characteristics.

Automated homecage systems enable continuous monitoring of various behavioral parameters, enhancing the throughput, validity, and reliability of rodent behavioral characterization [4]. These systems hold promise for advancing translational psychiatric research in rodents and facilitating the discovery of new treatment options [8,18,29–36]. Food-rewarded learning is a common focus of such studies; however, most longitudinal studies have been conducted in relatively small cages and typically assessed animals for only 1–2 hours per day [37–39]. To date, only one study has examined the behavior of schizophrenia model mice using the IntelliCage system, demonstrating impairments in cognitive functions such as learning and memory [9]. While the delay discount paradigm is commonly used for short durations (approximately 1 hour/day), this is the first system to apply the method in a large homecage setting over an extended period under almost handling-free conditions.

Originally it was Kook and co-workers who described a method for homecage testing of the delay discount paradigm to assess impulsivity in rats [18]. However, their setup used a standard rat cage with an operant panel, providing minimal space for the animals and limiting testing to two short sessions (1 hour each) per day, spaced 8 hours apart. Behavioral testing in a large, enriched cage over a prolonged period allows for ecologically relevant assessments under lower-stress conditions and with improved predictive validity [3,7]. To address these limitations, we developed the novel HomeManner system, incorporating environmental enrichment and minimizing animal-experimenter interactions that could otherwise influence behavior. This system benefits both animal welfare and experimenter efficiency. The HomeManner setup uniquely enables rats to exhibit a continuous, self-initiated feeding pattern to access all available rewards, rather than being restricted to short engagement periods. In our study, decision-making behavior was assessed using a two-dispenser choice assay, quantifying preferences for 3 versus 1 reward pellet. To validate our protocol, we recorded and analyzed multiple performance measures, many of which align with those reported in earlier studies [40–42].

In the HomeManner apparatus, unlike the Ambitus test results, most parameters were comparable at the group level. However, activity at the reward trays allowed classification of the animals into four subgroups/subsets. This subgroup-based analysis highlighted behavioral impairments in the Wisket animals. Unlike the findings of Koot et al. (2009), who studied Wistar rats, not all animals in our study learned to prefer the larger reward, even over an extended period. The differences may be attributable to the use of a small cage for a short testing duration in the earlier study. Animals in SG1 displayed activity at both reward trays, and most developed a preference for the larger reward. Despite the short distance between the trays (7 cm), some animals explored only one tray (SG2 and SG3; n = 3 each in both control and Wisket groups), while others did not explore the trays at all (SG4; control n = 1; Wisket n = 2). This phenomenon suggests that the large space of the HomeManner apparatus may inhibit focused exploration. However, all of the standard laboratory diet (10 g daily), placed near the pellet dispensers, was successfully consumed. Another possibility is the low activity level and/or motivation of both Wistar and Wisket animals, which has been previously reported [22,43]. Consequently, detailed analysis of choice rates in the delay discount paradigm could only be conducted in SG1, where the animals learned to prefer the large reward (LD) side. Consistent with earlier findings (Evenden & Ryan, 1996), these animals frequently chose both reward options (small delay [SD] vs. large delay [LD]), likely to monitor potential changes in reward availability. While an equal number of animals (n = 4) in each group learned to prefer the LD tray, the shortest delay caused a complete loss of LD preference in most animals, except for one control animal (N4; see 4th bar in the control group, Fig 6). This animal tolerated a 50-second delay, resulting in a significantly longer mean delay time in the control group. The short delay observed suggests high impulsivity in both groups under this paradigm, while the behavior of animal N4 may indicate rigidity as well. The accuracy was significantly lower on the SD side in Wisket animals compared to controls. Prematurity was notably high on the SD side in both groups compared to the LD side, and no significant differences were observed in the incorrect response ratio between the control and Wisket groups. Consequently, only a subset of these parameters indicated a higher level of impulsivity in the Wisket model rats.

Analysis of the SG1 group with “no” or 10-second delay revealed significantly longer eating times in Wisket rats compared to controls on both sides with “no” delay. In controls, eating time was shorter on the LD side than on the SD side, while in Wisket rats, eating time was longer on the LD side, which was accompanied by impaired learning capacity. Additionally, both groups exhibited shorter eating times and increased exploratory activity on the SD side compared to the LD side with a 10-second delay, reflecting changes in side preference. The introduction of a 10-second delay moderately decreased accuracy and significantly increased prematurity on the SD side compared to the LD side, particularly in Wisket animals.

The incorrect response ratio in control animals remained relatively stable, indicating their ability to distinguish specific time intervals when the panel was effectively available. In contrast, Wisket rats showed a significant increase in incorrect response ratio on both sides after the introduction of the 10-second delay, suggesting they failed to remain at rest while waiting for rewards and repeatedly checked both trays. These findings indicate impaired learning and heightened impulsivity in the SG1 subgroup of Wisket animals.

Spearman correlation analysis within SG1 showed a similar sensitivity to delays in both groups, suggesting that only some parameters were differentially affected by delays in control and Wisket animals.

For the six rats with activity limited to one side (SG2 and SG3), impulsivity could only be assessed using accuracy, prematurity, and incorrect response ratio. Alterations in these parameters, particularly in SG3, indicated a higher level of impulsivity in Wisket animals.

Interestingly, animals that explored only the LD side (SG2) completely ignored the delays, suggesting a high level of behavioral rigidity, consistent with previous findings [24]. The primary difference between the two groups in SG2 was the longer delay time observed in Wisket animals. SG3 animals consumed rewards exclusively from the SD trays and exhibited marked differences in most parameters compared to the SG1 and SG2 subgroups. Specifically, SG3 animals had shorter eating times and highly intense exploratory activity.

When comparing control and Wisket animals within SG3, Wisket rats displayed greater exploratory activity and learning capacity. However, Wisket animals also showed lower accuracy and higher prematurity, indicating impaired attention and/or heightened restlessness and impulsivity [44].

Individuality is a fundamental characteristic of living beings and is becoming an increasingly important factor in diagnosis and treatment. The high inter-individual variability in behavioral profiles observed in both humans and animals may arise from differences in age, sex, genetics, cognitive functions, and environmental factors [1,45]. Environmental enrichment may further increase inter-individual variability due to variations in experience and/or behavioral motives [1,46].

In preclinical research, achieving homogeneity and low variability is crucial to detect desired statistical effects. Consequently, large differences between individuals could be seen as a limitation of the HomeManner test. However, this approach may be better suited to mimic real-life conditions, and longitudinal, individualized screening can provide a detailed characterization of neuropsychiatric animal models. Personalized analyses could uncover novel mechanisms underlying mental disorders, suggesting that shifting focus from group patterns to individual outcomes might enhance translational relevance [47,48]. Preclinical studies increasingly emphasize the significance of individuality among animals. For instance, the Nestler group conducted an innovative study in which they stratified individual mice based on their performance in a social interaction test [49]. Similarly, another study classified genetically homogeneous mice into subpopulations based on their responses to antidepressant treatment [50].

In the present study, prolonged observation of animals in a large, environmentally enriched cage revealed the true individuality of their behaviors. While extended time in the HomeManner system reduced differences between the two groups, it amplified individual variability. Consequently, only analyses based on categorized data could uncover the behavioral impairments in Wisket animals.

Evidence suggests that environmental enrichment can mitigate schizophrenia-like symptoms in animal models [51–55]. For instance, administering an NMDA receptor antagonist to juvenile Long Evans female rats induced cognitive disturbances, which were alleviated by 18 days of adult exposure to environmental enrichment [52]. Each rat in this study displayed a unique behavioral profile, making average group patterns less informative, as observed in earlier research [18]. Detailed analysis revealed lower inter-individual variability in Wisket rats of SG1 compared to controls.

Further studies are needed to identify the factors—such as activity, emotion, and attention—that best explain performance differences. It is likely that all of these factors play a role, but their influence varies depending on individual circumstances [56].

In conclusion, while group-level analysis in the HomeManner system did not reveal significant differences between control and Wisket animals, detailed subgroup and individualized analyses identified impairments in several behavioral parameters in the Wisket group. These findings align with clinical observations, as schizophrenia, a heterogeneous disorder, is characterized by a wide range of symptoms that vary across patients and over time.

Although impaired cognition, often accompanied by impulsivity, is commonly observed in schizophrenia patients and animal models, these traits are not universally present. Some patients exhibit enhanced cognitive abilities and creativity in daily life while showing impaired cognition during acute or stressful testing conditions [57–59]. Schizophrenia is not strictly an impulse control disorder, but patients frequently report more impulsive behaviors, have higher rates of substance use, and exhibit dysfunction in brain circuits associated with impulsivity [17,60].

Despite the strengths of the HomeManner system, several limitations and areas for improvement remain. While the system was optimized for long-term use and designed to minimize damage from rats, animals occasionally chewed the dispenser sensors, disrupting the device’s functionality. Additionally, the system shares common limitations with all two-dispenser choice assays, such as positional bias, which can influence decision-making behavior.

Currently, the device is suitable only for the behavioral assessment of singly housed rats, which limits the number of simultaneous observations and introduces a degree of stress that could affect behavioral profiles [61]. Moreover, the mild food deprivation used to maintain task motivation may have introduced some stress. However, previous studies suggest that individual housing of adult rats does not significantly impact several stress-related parameters, including cognitive function, and that environmental enrichment in the large home cage may have mitigated these effects [62].

A key limitation of this study is that the HomeManner system is currently designed only to monitor exploratory and eating activities in rats. Additionally, not all animals could be included in the detailed behavioral analysis of exploratory activity at both trays. However, such animals should not be excluded from group-level analyses; instead, other behavioral tests or video analyses could provide reliable data on their behavior. We propose that inactive animals in the HomeManner system could be included in drug-treatment groups to evaluate the effects of specific compounds on this behavioral phenomenon.

Although pharmacological studies will require a larger sample size, these initial findings suggest that future experiments might benefit from tailored individual treatments for different subgroups. While none of the observed behaviors are specific to schizophrenia, the complex behavioral profile of the Wisket animals, combined with the model’s constructive and predictive validity, supports further improvements in the face validity of this triple-hit schizophrenia-like Wisket rat strain. While our earlier study showed, in line with human data, that male animals exhibit more severe schizophrenia-like symptoms [12], further studies are needed to explore sex-dependent differences in this long-term behavioral paradigm. Additionally, while the HomeManner system was designed to minimize experimenter interaction, suggesting a stress-free environment, this assumption should be validated by measuring physiological stress markers such as blood corticosterone levels in future investigations.

The high level of individual variability observed in this study underscores the importance of personalized characterization, akin to approaches used in human practice. Future advancements should focus on implementing image analysis of video recordings using sophisticated software with artificial intelligence techniques for more detailed behavioral characterization. Finally, refining the current homecage system and validating additional task protocols will be essential for advancing future research.

## Conclusion

In summary, the present study provides an extended behavioral profile of triple-hit schizophrenia-like Wisket rats over a prolonged period using the newly developed HomeManner system. This automated, experimenter-free approach offers a promising tool for studying complex behaviors in conjunction with pharmacological interventions that typically require multiple and long-term testing phases. Moreover, rather than relying solely on group averages and excluding outlier data points, we emphasize the importance of individual characterization and stratification to enhance the translational utility of preclinical research in animal models of schizophrenia.
